# Associations between sport and screen-entertainment with mental health problems in 5-year-old children

**DOI:** 10.1186/1479-5868-7-30

**Published:** 2010-04-21

**Authors:** Lucy J Griffiths, Marsha Dowda, Carol Dezateux, Russell Pate

**Affiliations:** 1 MRC Centre of Epidemiology for Child Health, UCL Institute of Child Health, London, UK; 2 Arnold School of Public Health, University of South Carolina, Columbia, South Carolina, USA

## Abstract

**Background:**

Few studies have examined the benefits of regular physical activity, and risks of sedentary behaviour, in young children. This study investigated associations between participation in sports and screen-entertainment (as components of physical activity and sedentary behaviour), and emotional and behavioural problems in this population.

**Methods:**

Cross-sectional analysis of data from 13470 children (50.9% boys) participating in the nationally representative UK Millennium Cohort Study. Time spent participating in sports clubs outside of school, and using screen-entertainment, was reported by the child's mother at child age 5 years, when mental health was also measured using the Strengths and Difficulties Questionnaire.

**Results:**

45% of children did not participate in sport clubs and 61% used screen-entertainment for ≥ 2 hours per day. Children who participated in sport had fewer total difficulties; emotional, conduct, hyperactivity-inattention and peer relationship problems; and more prosocial behaviours. These relationships were similar in boys and girls. Boys and girls who used screen-entertainment for any duration, and participated in sport, had fewer emotional and behavioural problems, and more prosocial behaviours, than children who used screen-entertainment for ≥ 2 hours per day and did not participate in sport.

**Conclusions:**

Longer durations of screen-entertainment usage are not associated with mental health problems in young children. However, our findings suggest an association between sport and better mental health. Further research based on longitudinal data is required to examine causal pathways in these associations and to determine the potential role of this and other forms of physical activity in preventing mental health disorders.

## Background

In Great Britain, ten percent of children (aged 5-16) have a mental health disorder such as hyperactivity, or an emotional (anxiety or depression) or conduct disorder [1]. These children are at risk of school exclusion, antisocial behaviour, offending, and drug and alcohol misuse, and are also prone to mental health problems later in life [2]. Poverty, abuse and parental separation are some of the known risk factors for social and emotional problems [3] but lifestyle factors that contribute to, or protect against, mental health disorders remain unclear. Identifying these factors may help to inform the development of effective mental health interventions.

Physical activity is important for psychological well-being [4,5] and has been found to reduce anxiety, depression and behavioural problems [6-8]. The majority of studies investigating associations between physical activity and mental health problems have focused on adults [4] and adolescents [7,9-16], with relatively few studies in young children. A recent study (mean age 8.5 years) [17] reported that psychological distress was associated with low levels of activity and higher levels of sedentary behaviour. Furthermore, the benefits of physical activity may be different in boys and girls; Sagatun et al [13] observed a significant association between physical activity and mental health in adolescent boys but not girls.

Research suggests that sedentary behaviours, like television viewing (TV) or computer use (hereafter termed screen-entertainment), are independent of physical activity involvement [18,19]. Despite this, sedentary behaviours have been linked to mental health problems in children, including attention [20,21] and behavioural conduct disorders [22,23]. Nevertheless, there is limited evidence of these associations in young children or of differences by sex. Furthermore, few studies have examined a range of mental health problems.

The aim of this study was to examine associations between participation in sports clubs or lessons, and screen-entertainment, and a range of emotional and behavioural problems in a nationally representative cohort of 5-year-old children. We also investigated whether these associations differed between boys and girls. We hypothesized that both girls and boys who did not participate in sport, who used screen-entertainment for ≥ 2 hours a day, or the combination of these behaviours, would have more emotional and behavioural problems.

## Methods

### Subjects and Design

The Millennium Cohort Study (MCS) is a prospective study of the social, economic and health-related circumstances of British children born between September 2000 and January 2002 who were resident in the United Kingdom and eligible for Child Benefit (a universal benefit for families with children). A stratified clustered sampling design was employed to over-represent children living in disadvantaged areas, from ethnic minority groups, and from Wales, Scotland and Northern Ireland. The first contact with the cohort was at 9 months, when information was collected on 18818 infants (18296 singletons), 72% of those approached [24]. The second and third surveys took place when the cohort were aged 3 and 5 years [25]. At each contact, survey interviews were conducted within the home and information obtained from main (usually the child's mother) and partner respondents. The MCS received ethical approval from the South West and London Multi-Centre Research Ethics Committees [25].

The MCS data were obtained from the UK Data Archive, University of Essex, UK. For this analysis, we examined data from the first and third contacts, which included 14403 singleton children. However, we excluded families in which the main survey respondent was not female (n = 370) or the child's biological mother (n = 41), where there were two cohort children from the same family (n = 9), or if data for sports participation (n = 29), screen-entertainment (n = 36) or any of the emotional or behavioural problems (n = 522) were missing. Some participants had more than one exclusion criterion, resulting in a sample size of 13470 children. Children were more likely to be excluded if they were from an ethnic minority group, if they were obese, if their mothers were unemployed or had lower academic qualifications, socioeconomic circumstances or household incomes, if they had lone mothers, or if there were more than two children in their household; however, the overall differences between those included and excluded were small (data not shown).

### Predictor variables

At age 5, mothers reported how many days a week their child went to a club or class to do sport or any other physical activity outside of school lessons, like swimming, gymnastics, football or dancing. Responses ranged from none to five or more days/week. A physical activity variable was derived based on whether the child participated in any sport clubs or lessons (i.e. on 1 - 5 days/week) or less often than 1 day/not at all. Mothers were also asked how many hours a day their child watched television/videos/DVDs and used a computer or played electronic games. A sedentary activity variable was derived based on whether the child used screen-entertainment for ≥ 2 hours or < 2 hours per day. This 2-hour cut-off was based on the American Academy of Pediatrics recommended threshold for daily media time[26]. Finally, these two derived variables were combined to examine both sport and screen-entertainment. Children were classified into one of four groups: i) no participation in sports and using screen-entertainment for ≥ 2 hours, ii) participation in sports and using screen-entertainment for ≥ 2 hours, iii) no participation in sports and using screen-entertainment for < 2 hours, or iv) participation in sports and using screen-entertainment for < 2 hours.

### Outcome variables

At age 5, parents completed the Strengths and Difficulties Questionnaire (SDQ) [27].

The SDQ is an emotional and behavioural screening questionnaire consisting of 25 psychological attributes. These are divided between five scales which examine: emotional symptoms (complains of headaches/stomach aches/sickness, often worried, often unhappy, nervous or clingy in new situations, easily scared); conduct problems (often has temper tantrums, *generally obedient*, fights with or bullies other children, can be spiteful to others, often argumentative with adults); hyperactivity/inattention problems (restless, overactive, cannot stay still for long, constantly fidgeting, easily distracted, *can stop and think before acting*, *sees tasks through to end*); peer relationship problems (tens to play alone, *has at least one good friend*, *generally liked by other children*, picked on or bullied by other children, gets on better with adults); and pro-social behaviour (considerate of others' feelings, shares readily with others, helpful if someone is hurt or ill, kind to younger children, often volunteers to help others).

Parents score the items as 'not true', 'somewhat true' and 'certainly true', with responses coded as 0, 1 and 2, respectively (those in italics are reverse scored). Scores range from 0 to 10; higher scores indicate more problems, with the converse for higher scores on the pro-social scale. A 'total difficulties' score is derived from the sum of scores for the emotional, conduct, hyperactivity and peer relationship problem scales; scores range from 0 to 40 with higher scores also indicating more difficulties. Recommended cutoff scores are available to identify children who are 'likely cases' with mental health disorders [27] but this study used the scales as continuous variables.

The SDQ has been used in other large epidemiological studies, such as the British Child Mental Health Survey and the Avon Longitudinal Study of Parents and Children [28,29]. It is also reported to have high test-retest reliability and good validity [30].

### Potential confounding factors

A range of social, demographic and health information has been collected on the cohort children and their families. The following factors, collected at the first and third contacts, were examined as potential confounding factors: collected at the first contact (child age: 9 months) - child's gender, ethnicity (categorised according to guidelines from the Office for National Statistics [31]), maternal socio-economic status (classified according to the National Statistics Socio-economic Classification [32]), and the mothers highest academic qualification attained; collected at the third contact (child age: 5 years) - maternal employment, household income, lone motherhood status, number of siblings in household, maternal and child longstanding illnesses, and maternal emotional problems (e.g. feeling anxious or depressed in the last month). Weight status at age 5 was also considered as a potential confounding factor. At age 5, children were weighed using Tanita HD-305 scales (Tanita UK Ltd, Middlesex, UK), recorded to the nearest 0.1 kg, and height was measured using Leicester Height Measure Stadiometers (Seca Ltd, Birmingham, UK), recorded to the nearest 0.1 cm. Childhood overweight (including obesity) was defined using the International Obesity Task Force cut-off for BMI [33]. Those below this cut-off were classified as 'normal weight'.

### Statistics

Analyses were conducted in STATA/SE 10.0 (Stata Corporation, TX), using survey commands to allow for the cluster sampling design and to obtain robust standard errors. Weighted percentages, univariate and adjusted analyses were calculated using survey and non-response weights to account for the sampling design and attrition between contacts.

Relationships between frequency of participation in sports clubs or classes, hours of screen-entertainment and combined sport/screen-entertainment with each of the five SDQ scales (emotional symptoms, conduct problems, hyperactivity/inattention, peer relationship problems, and pro-social behaviour) and the composite SDQ total score were investigated using linear regression analyses. Coefficients, their 95% confidence intervals and p-values are reported. Univariate models were adjusted for factors found to be significantly associated with both the predictor and outcome variables within the model as they were therefore considered to be confounding factors. Stratified gender analyses were conducted.

## Results

### Description of the sample

The mean age of the children was 5.2 years when, in the UK, nearly all children are in school. The characteristics of all children, and boys and girls separately, are provided in Table 1.

**Table 1:** Sample Characteristics

Characteristic	All children	Boys	Girls
	N = 13470	N = 6883	N = 6587
	**% or Mean (SD) (n)**		

**Ethnicity**			
White	88.9 (11671)	89.0 (5973)	88.9 (5698)
Mixed	3.1 (373)	3.0(180)	3.2 (193)
Asian	4.6 (899)	4.5 (453)	4.7 (446)
Black	2.3 (353)	2.5 (186)	2.2 (167)
Other	1.0 (145)	1.0 (76)	1.0 (69)
**Weight status^a^**			
Normal weight	79.0 (10394)	81.2 (5456)	76.7 (4938)
Overweight	15.7 (2143)	13.9 (965)	17.5 (1178)
Obese	5.3 (742)	4.9 (350)	5.7 (392)
**Maternal employment**			
Full time	12.1 (1729)	12.4 (898)	11.8 (831)
Part time	38.2 (4924)	38.0 (2524)	38.3 (2400)
On leave	2.8 (391)	2.9 (201)	2.6 (190)
Self-employed	6.3 (786)	6.4 (394)	6.3 (392)
Unemployed or Student	40.6 (5617)	40.2 (2855)	41.0 (2762)
**Maternal highest qualification**			
Degree	18.2 (2384)	17.9 (1195)	18.5 (1189)
Diploma	9.6 (1260)	9.9 (657)	9.4 (603)
A/AS/S levels	9.9 (1354)	9.6 (684)	10.1 (670)
O level/GCSE grades a-c	35.8 (4666)	36.2 (2414)	35.4 (2252)
GCSE grades d-g	10.7 (1414)	10.8 (741)	10.7(673)
Other	1.9 (287)	1.9 (133)	2.0 (154)
None	13.9 (2078)	13.8 (1041)	14.0 (1037)
**Maternal socio-economic status**			
Managerial & professional	31.2 (3948)	31.0 (2001)	31.4 (1947)
Intermediate	18.5 (2374)	18.4 (1221)	18.7 (1153)
Small employers & own	4.3 (501)	4.4 (265)	4.0 (236)
Lower supervisor & tech	5.5 (766)	5.5 (370)	5.5 (396)
Semi-routine & routine	34.7 (4762)	35.2 (2465)	34.2 (2297)
Never worked/unemployed	5.8 (962)	5.4 (473)	6.2 (489)
**Household Income (€)**			
0-12999 pa	21.6 (3132)	21.4 (1576)	21.9 (1556)
13000-25999 pa	32.7 (4556)	32.8 (2346)	32.6 (2210)
26000-36399 pa	19.4 (2504)	19.3 (1279)	19.5 (1225)
≥ 36400 pa	26.3 (3164)	26.5 (1619)	26.0 (1545)
**Lone motherhood status**			
Non-lone mother	81.0 (10867)	81.0 (5549)	81.1 (5318)
Lone mother	19.0 (2603)	19.0 (1334)	18.9 (1269)
**No. siblings in household**			
1 child	16.4 (2227)	16.2 (1140)	16.5 (1087)
2 children	49.4 (6447)	49.5 (3285)	49.2 (3162)
>2 children	34.3 (4796)	34.2 (2458)	34.3 (2338)
**Child: Longstanding illness**			
Yes	19.4 (2623)	22.0 (1520)	16.7 (1103)
No	80.6 (10840)	78.0 (5361)	83.3 (5479)
**Mother: Longstanding illness**			
Yes	23.9 (3238)	23.4 (1626)	24.5 (1612)
No	76.1 (10230)	76.6 (5255)	75.5 (4975)
**Maternal emotional problems**			
No	48.9 (6836)	49.4 (3500)	48.3 (3336)
Yes	51.1 (6631)	50.6 (3380)	51.7 (3251)

Missing data (all children): gender 0; ethnicity 29; weight status 191; maternal employment 23; maternal highest qualification 27; maternal socio-economic status 157; household Income 114; lone motherhood status 0; no. siblings in household 0; child - longstanding illness 7; mother - longstanding illness 2; maternal emotional problems 3.

^a ^Defined using the International Obesity Task Force cut-offs for BMI[33].

### Participation in sports clubs or classes and hours of screen-entertainment

44.6% of children did not participate in sport outside of school (Table 2), with boys (48.3%) significantly less likely to take part than girls. 60.7% of children used screen-entertainment for ≥ 2 hours each day, with boys (64.9%) significantly more likely to do so than girls. 32.5% of boys and 24.2% of girls did not participate in sport *and *used screen-entertainment for ≥ 2 hours each day, i.e. were among the most sedentary.

**Table 2:** Sport, screen-entertainment and emotional and behavioural problems scores

Characteristic	All children	Boys	Girls
	% or Mean (SD) (n)		
**Participation in any sports clubs**			
No	44.6 (6277)	48.3 (3448)	40.8 (2829) *
Yes	55.4 (7193)	51.7 (3435)	59.2 (3758) *
**Hours screen-entertainment per day**			
< 2 hours	39.3 (5249)	35.1 (2366)	43.7 (2883) *
≥ 2 hours	60.7 (8221)	64.9 (4517)	56.3 (3704) *
**Sports and screen-entertainment (SE)**			
No sports and ≥ 2 hours SE	28.5 (4004)	32.5 (2342)	24.2 (1662) *
Sports and ≥ 2 hours SE	32.2 (4217)	32.4 (2175)	32.1 (2042)
No sports and < 2 hours SE	16.2 (2273)	15.8 (1106)	16.6 (1167)
Sports and < 2 hours SE	23.2 (2976)	19.4 (1260)	27.1 (1716) *
SDQ ** scores			
Emotional problems	1.3 (1.6)	1.3 (1.6)	1.4 (1.6)
Conduct problems	1.5 (1.5)	1.6 (1.6)	1.3 (1.4) *
Hyperactivity/inattention problems	3.3 (2.4)	3.6 (2.4)	2.9 (2.2) *
Peer relationship problems	1.1 (1.4)	1.2 (1.5)	1.0 (1.3) *
Prosocial behaviours	8.4 (1.7)	8.1 (1.8)	8.7 (1.5) *
Total difficulties	7.2 (5.0)	7.7 (5.2)	6.7 (4.7) *

*Significant difference (P ≤ 0.05) between boys and girls.

** Strengths and Difficulties Questionnaire

### Emotional and behavioural problems

Boys had significantly more conduct problems, hyperactivity/inattention problems, peer relationship problems, and total difficulties (Table 2). Girls had significantly more pro-social behaviours.

### Regression analyses

#### Participation in sports clubs or classes

Both boys and girls who participated in sport had significantly fewer total difficulties (Table 3). They also had fewer emotional problems, conduct problems, hyperactivity-inattention problems, peer relationship problems and more pro-social behaviours. These associations remained significant after controlling for confounding factors, with the exception of hyperactivity-inattention problems in boys.

**Table 3:** Associations between any sports (yes versus no (referent)) and SDQ scales: regression coefficients and 95% confidence intervals.

	Emotional^a^	Conduct^b^	Hyperactivity^a^	Peer^a^	Prosocial^c^	Total^a^
**Univariable Results**						
Boys	-0.39 (-0.46, -0.31); p < 0.001	-0.48 (-0.60, -0.39); p < 0.001	-0.55 (-0.69, -0.41); p < 0.001	-0.49 (-0.55, -0.42); p < 0.001	0.22 (0.12, 0.32); p < 0.001	-1.89 (-2.16, -1.63); p < 0.001
Girls	-0.52 (-0.62, -0.43); p < 0.001	-0.50 (-0.59, -0.42); p < 0.001	-0.81 (-0.93, -0.69); p < 0.001	-0.45 (-0.53, -0.37); p < 0.001	0.21 (0.13, 0.30); p < 0.001	-2.29 (-2.56, -2.01); p < 0.001

**Adjusted results**						
Boys	-0.18 (-0.26, -0.10); p < 0.001	-0.14 (-0.22, -0.05); p = 0.002	-0.06 (-0.20, 0.07); p = 0.35	-0.21 (-0.29, -0.13); p < 0.001	0.11 (0.00, 0.21); p = 0.04	-0.60 (-0.86, -0.33); p < 0.001
Girls	-0.31 (-0.41, -0.21); p < 0.001	-0.19 (-0.28, -0.11); p < 0.001	-0.33 (-0.45, -0.20); p < 0.001	-0.18 (-0.25, -0.10); p < 0.001	0.11 (0.01, 0.20); p = 0.03	-1.02 (-1.29, -0.75); p < 0.001

Regression coefficients represent differences in SDQ scores compared with the reference group.

^a ^Adjusted for gender, child's ethnicity, maternal employment status, mother's highest academic qualification, maternal socio-economic status, household income, lone parent status, no. of children in household, child's longstanding illness, mother's longstanding illness, maternal emotional problems.

^b ^Adjusted for gender, maternal employment status, mother's highest academic qualification, maternal socio-economic status, household income, lone parent status, no. of children in household, child's longstanding illness, mother's longstanding illness, maternal emotional problems.

^c ^Adjusted for gender, maternal employment status, mother's highest academic qualification, maternal socio-economic status, household income, lone parent status, no. of children in household, child's longstanding illness, maternal emotional problems.

#### Screen-entertainment

Screen-entertainment was not significantly associated with any problems in girls, whereas boys who used screen-entertainment for < 2 hours per day had significantly fewer conduct disorders (Table 4). After controlling for confounding factors, only two significant associations were observed: girls who used screen-entertainment for <2 hours per day had more emotional problems and more conduct problems.

**Table 4:** Associations between hours of screen-entertainment (< 2 hours versus ≥ 2 hours (referent)) and SDQ scales: regression coefficients and 95% confidence intervals.

	Emotional^a^	Conduct^b^	Hyperactivity^b^	Peer^b^	Prosocial^a^	Total^b^
**Univariable Results**						
Boys	-0.06 (-0.15, 0.02); p = 0.16	-0.10 (-0.19, -0.01); p = 0.03	-0.02 (-0.17, 0.13); p = 0.81	-0.07 (-0.15, 0.02); p = 0.14	-0.01 (-0.11, 0.10); p = 0.92	-0.25 (-0.56, 0.06); p = 0.12
Girls	0.05 (-0.03, 0.13); p = 0.23	0.01 (-0.08, 0.09); p = 0.83	-0.11 (-0.23, 0.02); p = 0.11	-0.07 (-0.15, 0.01); p = 0.08	-0.02 (-0.10, 0.07); p = 0.73	-0.12 (-0.39, 0.15); p = 0.40

**Adjusted Results**						
Boys	-0.03 (-0.10, 0.07); p = 0.77	-0.01 (-0.10, 0.08); p = 0.78	0.11 (-0.03, 0.26); p = 0.12	-0.00 (-0.09, 0.09); p = 0.94	-0.04 (-0.14, 0.06); p = 0.45	0.08 (-0.22, 0.37); p = 0.60
Girls	0.09 (0.01, 0.18); p = 0.03	0.09 (0.01, 0.17); p = 0.03	0.04 (-0.09, 0.16); p = 0.55	-0.02 (-0.09, 0.06); p = 0.68	-0.05 (-0.13, 0.03); p = 0.21	0.19 (-0.05, 0.44); p = 0.13

Regression coefficients represent differences in SDQ scores compared with the reference group.

^a ^Adjusted for gender, maternal employment status, mother's highest academic qualification, maternal socio-economic status, household income.

^b ^Adjusted for gender, child's weight status, maternal employment status, mother's highest academic qualification, maternal socio-economic status, household income.

#### Sports and screen-entertainment

Less than 2 hours screen-entertainment and sports versus ≥ 2 hours screen-entertainment and no sports (i.e. most active versus most sedentary): Boys and girls had fewer emotional problems, conduct problems, hyperactivity-inattention problems, peer relationship problems and total difficulties, and more pro-social behaviours (Table 5). Following adjustment for confounding factors, boys had fewer emotional problems, conduct problems, peer relationship problems and total difficulties. Girls had fewer emotional problems, hyperactivity-inattention problems, peer relationship problems and total difficulties.

**Table 5:** Associations between hours of screen-entertainment and sports and SDQ scales: regression coefficients and 95% confidence intervals.

	Emotional^a^	Conduct^b^	Hyperactivity^c^	Peer^c^	Prosocial^d^	Total^c^
**Univariable Results**						
Boys						
<2 hr/sports	-0.44 (-0.54, -0.34); p < 0.001	-0.56 (-0.67, -0.44); p < 0.001	-0.53 (-0.74, -0.33); p < 0.001	-0.53 (-0.64, -0.42); p < 0.001	0.21 (0.08, 034); p = 0.002	-2.06 (-2.44, -1.68); p < 0.001
<2 hr/no sports	0.02 (-0.12, 0.16); p = 0.8	-0.04 (-0.19, 0.11); p = 0.6	-0.02 (-0.26, 0.22); p = 0.9	-0.00 (-0.14, 0.14); p = 0.9	-0.03 (-0.20, 0.14); p = 0.7	-0.04 (-0.55, 0.47); p = 0.9
≥ 2 hr/sports	-0.35 (-0.45, -0.24); p < 0.001	-0.45 (-0.55, -0.34); p < 0.001	-0.57 (-0.73, -0.40); p < 0.001	-0.46 (-0.55, -0.37); p < 0.001	0.22 (0.10, 0.33); p < 0.001	-1.81 (-2.14, -1.48); p < 0.001
≥ 2 hr/no sports	0	0	0	0	0	0
Girls						
<2 hr/sports	-0.45 (-0.57, -0.33); p < 0.001	-0.47 (-0.58. -0.35); p < 0.001	-0.87 (-1.03, -0.70); p < 0.001	-0.49 (-0.60, -0.39); p < 0.001	0.19 (0.07, 0.30); p = 0.002	-2.28 (-2.66, -1.90); p < 0.001
<2 hr/no sports	0.06 (-0.08, 0.20); p = 0.4	0.08 (-0.06, 0.22); p = 0.3	0.15 (-0.05, 0.36); p = 0.1	-0.01 (-0.15, 0.12); p = 0.9	-0.07 (-0.21, 0.06); p = 0.3	0.28 (-0.14, 0.71); p = 0.2
≥ 2 hr/sports	-0.54 (-0.66, -0.41); p < 0.001	-0.47 (-0.58, -0.37); p < 0.001	-0.65 (-0.81; -0.50); p < 0.001	-0.42 (-0.51, -0.33); p < 0.001	0.18 (0.07, 0.29; p = 0.002	-2.08 (-2.42, -1.75); p < 0.001
≥ 2 hr/no sports	0	0	0	0	0	0

**Adjusted results**						
Boys						
<2 hr/sports	-0.20 (-0.32, -0.09); p < 0.001	-0.16 (-0.28, -0.03); p = 0.01	0.06 (-0.15, 0.27); p = 0.6	-0.22 (-0.34, -0.09); p < 0.001	0.07 (-0.07, 0.21); p = 0.3	-0.54 (-0.94, -0.13); p = 0.01
<2 hr/no sports	0.05 (-0.08, 0.18); p = 0.5	0.01 (-0.13, 0.15); p = 0.9	0.04 (-0.19, 0.26); p = 0.8	0.02 (-0.11, 0.16); p = 0.7	-0.06 (-0.23, 0.11); p = 0.5	0.11 (-0.36, 0.58); p = 0.6
≥ 2 hr/sports	-0.15 (-0.27, -0.04); p = 0.01	-0.12 (-0.22, -0.02); p = 0.02	-0.11 (-0.27, 0.06); p = 0.2	-0.21 (-0.3, -0.11); p < 0.001	0.10 (-0.02, 0.22); p = 0.1	-0.60 (-0.93, -0.27); p < 0.001
≥ 2 hr/no sports	0	0	0	0	0	0
Girls						
<2 hr/sports	-0.20 (-0.32, -0.08); p = 0.001	-0.09 (-0.20, 0.02); p = 0.1	-0.27 (-0.43, -0.11); p = 0.001	-0.20 (-0.30, -0.09); p < 0.001	0.04 (-0.08, 0.16); p = 0.5	-0.79 (-1.14, -0.44); p < 0.001
<2 hr/no sports	0.08 (-0.06, 0.21); p = 0.3	0.13 (-0.01, 0.27); p = 0.07	0.22 (0.01, 0.42); p = 0.04	-0.03 (-0.17, 0.12); p = 0.7	-0.10 (-0.24, 0.04); p = 0.2	0.37 (-0.05, 0.79); p = 0.09
≥ 2 hr/sports	-0.34 (-0.47, -0.21); p < 0.001	-0.18 (-0.28, -0.07); p = 0.001	-0.21 (-0.37, -0.06); p = 0.01	-0.19 (-0.29, -0.10); p < 0.001	0.07 (-0.04, 0.19); p = 0.2	-0.94 (-1.28, -0.61); p < 0.001
≥ 2 hr/no sports	0	0	0	0	0	0

Regression coefficients represent differences in SDQ scores compared with the reference group (≥ 2 hr/no sports).

^a ^Adjusted for gender, child's ethnicity, maternal employment status, mother's highest academic qualification, maternal socio-economic status, household income, lone parent status, no. of children in household, mother's longstanding illness, maternal emotional problems.

^b ^Adjusted for gender, child's weight status, maternal employment status, mother's highest academic qualification, maternal socio-economic status, household income, lone parent status, no. of children in household, mother's longstanding illness, maternal emotional problems.

^c ^Adjusted for gender, child's ethnicity, child's weight status, maternal employment status, mother's highest academic qualification, maternal socio-economic status, household income, lone parent status, no. of children in household, mother's longstanding illness, maternal emotional problems.

^d ^Adjusted for gender, maternal employment status, mother's highest academic qualification, maternal socio-economic status, household income, lone parent status, no. of children in household, maternal emotional problems.

Less than 2 hours screen-entertainment and no sports versus ≥ 2 hours screen-entertainment and no sports (i.e. effect of screen entertainment in physically inactive): There were no significant differences for boys and girls for any of the emotional and behavioural problems within the univariate analyses (Table 5), or for boys in the adjusted analyses. Girls had more hyperactivity-inattention problems.

More or equal to 2 hours screen-entertainment and sports versus ≥ 2 hours screen-entertainment and no sports (i.e. effect of physical activity in high screen entertainment users): Boys and girls had fewer emotional problems, conduct problems, hyperactivity-inattention problems, peer relationship problems and total difficulties, and more pro-social behaviours (Table 5). Following adjustment, boys had fewer emotional problems, conduct problems, peer relationship problems and total difficulties. Girls had fewer emotional problems, conduct problems, hyperactivity-inattention problems, peer relationship problems and total difficulties.

## Discussion

### Summary of findings

Children who participate in sports clubs or classes outside of school exhibit lower total difficulties scores, fewer emotional, conduct, hyperactivity-inattention, and peer relationship problems and more prosocial behaviours. However, 45% of children in this UK-wide cohort did not participate in any sport outside of school, while 29% did not participate in any sport *and *used screen-entertainment >2 hours per day. Although 61% of children used screen-entertainment for >2 hours per day, this behaviour was only associated with emotional and conduct problems in girls, but not with any of the other mental health outcomes.

Boys and girls who participated in sport had fewer mental health problems, and more prosocial behaviours irrespective of the duration of their daily screen-entertainment use.

### Strengths and limitations

The breadth of information collected within the MCS permitted the exploration of associations between an indicator of physical activity, and sedentary behaviour, and mental health in a contemporary, nationally representative, cohort of young children. Furthermore, the depth of information collected on social and demographic factors allowed us to control for a range of confounding factors, thereby reducing the potential for residual confounding due to unmeasured individual characteristics. Although information on screen-entertainment and sports participation were via parental (proxy) report that were not validated against objective or observational measures of these activities, information on other health behaviours and information collected within the MCS has been shown to be valid and reliable [34-36]. The SDQ is also a valid and reliable instrument for assessing mental health problems in children [37] and one that correlates with other clinical measures [30]. The parent-proxy version of this instrument used within this study was specifically developed for this age group and its usage therefore also overcomes limitations of previous studies that have used adult screening tools when examining young people.

The main limitation of this study is the cross-sectional study design, prohibiting the direction of causality to be inferred. Few longitudinal studies in childhood have been conducted in this area, although in adolescence higher activity levels are related to fewer mental health problems later on in life [12,13]. At the time of writing, information on physical activity, sedentary behaviours, and mental health problems had only been collected at age 5 in this cohort. This analysis forms a basis for a planned longitudinal analysis of data from the follow-up of this cohort. This will be enhanced by objective measurements of physical activity that have recently been collected from these children at age eight years.

### Comparison with other findings

The mean SDQ scores obtained in this study were similar to scores obtained from 5855 5-10 year olds in a 1999 survey of British children [38]. In common with the latter survey, our findings showed that, in general, boys had more problems/difficulties than girls and displayed less prosocial behaviour.

Following adjustment, longer hours of screen-entertainment were not associated with mental health problems in boys or girls. In girls, we observed that lower screen entertainment use was associated with more emotional and conduct problems, and with more hyperactivity-inattention problems if they did not participate in sports. To our knowledge, lower levels of screen-entertainment exposure have not previously been shown to be associated with poorer mental health, and these findings need to be confirmed within a longitudinal analysis as discussed above. Other studies have shown detrimental influences of screen-entertainment. For example, Mistry et al [23] reported that TV viewing for more than 2 hours was associated with fewer social skills, although not associated with behavioural outcomes, at 5.5 years of age. Furthermore, Hamer et al [17] reported that higher levels of TV increased total difficulties on the SDQ. Discrepancies between our findings and previous research may be due to the use of differing measures of mental health, adjustment for different confounding factors, or due to the age of the participants. Further research is required in this area to develop understanding of the possible, if any, adverse effect of these sedentary behaviours, which are so prevalent among children and young people. This research should include investigation of the timing and content of this media exposure, which may be mediating factors.

Our study has clearly shown statistically significant inverse associations between participation in sport and mental health problems: Children who took part in sport beyond the school setting had fewer emotional, conduct, peer and hyperactivity/inattention problems, fewer total difficulties, and more pro-social behaviours. These findings support existing evidence in adolescents showing that those who are active have fewer emotional, behavioural or social problems, [14,39] fewer total difficulties (also measured with the SDQ by Ussher et al [10]), fewer depressive symptoms, [40] and lower levels of anxiety [39]. Furthermore, this study is consistent with Hamer et al's [17] study in Scottish children, which showed that low activity levels were associated with more total difficulties. This study extends existing knowledge in this area by focusing on young children, by examining a range of emotional and behavioural problems, and by examining gender differences. Although some gender differences were observed for screen-entertainment, both boys and girls had fewer problems and more prosocial behaviours if they participated in sport.

Previous studies have identified significant gender differences in activity and sedentary behaviours, with many studies reporting higher activity levels in boys [41,42]. Although girls in this study participated in more sport than boys, there may be more out-of-school classes available for girls of this age in the UK, such as dance and gymnastic classes. Young boys may spend more time participating in 'free play' e.g. playing with a ball, which may not have been captured in the MCS question.

### Implications for policy and further research recommendations

It is currently recommended that all young people participate in physical activity of at least moderate intensity for one hour per day, and that, at least twice a week, this should include weight-bearing activities that produce high physical stresses to improve bone health, muscle strength, and flexibility [43]. Physical education (PE), exercise, sport, and other daily activities, in and out of school, all contribute to these overall activity levels. It is estimated that 96% of primary schools achieve high rates of participation in two hours of PE and school sport per week [44], and our findings show that over half of all 5-year-olds (55%) also participate in structured activities outside of school. These figures are encouraging given that this study has shown, for example, that girls, with an overall mean score of 6.7 on total difficulties, have a 1.02 reduction in this score if they participate in sport. Nevertheless, more young people still need to be supported to engage in regular physical activity and our study therefore supports public health recommendations, interventions, and policies to increase activity levels, such as the recent NICE physical activity guidelines [45]. Clearly, opportunities should also be appropriate for the children's age group to foster increased activity levels, physical and social skills, and for enjoyment.

Nine in 10 children in the UK have a computer at home, almost all 5-16 year olds have access to multi-channel TV, and eight in ten 5-16 year olds have their own TV [46]. The American Academy of Pediatrics recommends that parents should limit their children's total media time to no more than 1 to 2 hours per day [47]. There are currently no recognized screen-entertainment guidelines in the UK, and, although this study has not shown many associations with mental health problems, prolonged periods of engagement in these sedentary activities are discouraged as they may be associated with other adverse child health outcomes, such as obesity [48].

Psychological (e.g. improvements in body satisfaction, self-esteem, self-efficacy and social support) [49] and physiological (e.g. monoamine and endorphin hypotheses) [14] mechanisms are proposed to underlie the mental health benefits resulting from physical activity involvement. Further longitudinal cohort studies and randomized controlled trials are required to examine these and other causal pathways mediating associations between physical activity/inactivity and mental health. This research also should aim to identify which components of physical activity (e.g. timing of exposure, amount and intensity, and type) are most beneficial for mental health prevention, and treatment, programs for young children.

## Conclusions

The findings of this study suggest an association between sport and better mental health. Given these and other well known benefits of physical activity, public health interventions and policies should continue to increase opportunities for young people to engage in regular habitual physical activity, exercise, and sport. However, further research is needed, based on longitudinal data, to examine causal pathways in the associations between this behaviour and emotional/behavioural problems.

## Competing interests

The authors declare that they have no competing interests.

## Authors' contributions

LJG designed the study, analysed and interpreted the data and drafted the manuscript. MD participated in the data analyses, data interpretation and drafting of the manuscript. CD and RP participated in the design of the study, data interpretation and critically revised the manuscript. All authors have read and approved the final manuscript.
